# Lost in Classification: Lower Cognitive Functioning in Apparently Cognitive Normal Newly Diagnosed RRMS Patients

**DOI:** 10.3390/brainsci9110321

**Published:** 2019-11-13

**Authors:** Marco Pitteri, Stefano Ziccardi, Caterina Dapor, Maddalena Guandalini, Massimiliano Calabrese

**Affiliations:** Neurology section, Department of Neurosciences, Biomedicine and Movement Sciences, University of Verona, 37134 Verona, Italy; stefano.ziccardi@univr.it (S.Z.); caterina.dapor@univr.it (C.D.); maddalena.guandalini@univr.it (M.G.)

**Keywords:** multiple sclerosis, cognitive impairment, diagnosis, neuropsychological assessment

## Abstract

Cognitive functioning in multiple sclerosis (MS) patients is usually related to the classic, dichotomic classification of impaired vs. unimpaired cognition. However, this approach is far from mirroring the real efficiency of cognitive functioning. Applying a different approach in which cognitive functioning is considered as a continuous variable, we aimed at showing that even newly diagnosed relapsing–remitting MS (RRMS) patients might suffer from reduced cognitive functioning with respect to a matched group of neurologically healthy controls (HCs), even if they were classified as having no cognitive impairment (CI). Fifty newly diagnosed RRMS patients and 36 HCs were tested with an extensive battery of neuropsychological tests. By using Z-scores applied to the whole group of RRMS and HCs together, a measure of cognitive functioning (Z-score index) was calculated. Among the 50 RRMS patients tested, 36 were classified as cognitively normal (CN). Even though classified as CN, RRMS patients performed worse than HCs at a global level (*p* = 0.004) and, more specifically, in the domains of memory (*p* = 0.005) and executive functioning (*p* = 0.006). These results highlight that reduced cognitive functioning can be present early in the disease course, even in patients without an evident CI. The current classification criteria of CI in MS should be considered with caution.

## 1. Introduction

Multiple Sclerosis (MS) is one of the most common inflammatory neurodegenerative disorders of the human central nervous system (CNS), characterized histologically by multifocal areas of inflammation, demyelination, and neurodegeneration [1] within the white matter (WM) [2] as well as within cortical and deep gray matter (GM) [3].

In addition to physical disability, cognitive impairment (CI) is common in MS patients, with frequencies ranging from 43% to 70% [4] depending on the studied population, the tests used, and the applied cut-off scores [5,6]. CI can occur early in the disease course [7] and has been strongly associated with both focal and diffuse GM damage [8,9] and WM lesion measures [10,11]. The mainly affected cognitive domains are verbal learning and memory, attention, information processing speed, and executive functions [12]. CI can alter MS patients’ behavior and quality of life [13,14], leading to social and personal difficulties, despite minimal physical disability [15]. Longitudinal studies have shown that CI detected at the time of diagnosis can predict the conversion from clinically isolated syndrome to definite MS [16], the progression of physical disability [17], the transition to the secondary progressive (SP) phase [18], and the worsening of physical disability and GM atrophy in the long term [19]. These studies suggest that assessing cognitive functioning since the early phases of the disease is of paramount importance [20,21].

Despite the different batteries of neuropsychological tests used to assess cognitive functioning, the classification of CI is undoubtedly affected by the chosen cut-off applied [6,22] and by the number of neuropsychological tests used. Usually, MS patients are classified as having either “normal cognition” or “impaired cognition” in a perspective of dichotomous classification (unimpaired vs. impaired). This approach, however, is far from being meaningful considering the real life [5], in which measures of functional aspects, such as cognitive functioning, resemble continuous variables, as also underlined in other neurological populations (e.g., see [23,24]).

Dichotomizing continuous variables, such as cognitive functions, carries the risk of losing information that might increase the number of false positive results, as well as of underestimating the extent of variation in patients’ performance [25], rendering difficult the diagnosis and the subsequent clinical decisions. For this reason, it would be more appropriate to use different psychometric methods, switching from a “cognitive impairment-based” to a “cognitive functioning-based” approach, considering cognitive functioning as a continuum variable as it is in real life, ranging from a minimum to a maximum level of performance. This is of particular interest given that cognitive decline may develop as a result of gradual progression, related to neurodegeneration and brain atrophy, or of acute disease activity, for which decline in cognitive performance can be often followed by incomplete recovery, thus contributing to the burden of CI in the long term [11].

In order to investigate the usefulness of this approach, with the present study we aimed at investigating the cognitive performance of a group of newly diagnosed MS patients with relapsing–remitting (RR) course as compared to a group of healthy controls (HCs). We expected that also the newly diagnosed MS patients, even if classified as being “cognitively normal” when referring to the classic, dichotomous approach, would rather show reduced cognitive functioning with respect to HCs.

## 2. Materials and Methods

### 2.1. Participants

Fifty consecutive newly diagnosed RRMS patients (37 females, mean ± SD age = 38.2 ± 11.6 years; mean ± SD education = 14.2 ± 2.7 years; mean ± SD disease duration from onset = 3.5 ± 5.2 years; median [range] effects of disability (EDSS) = 1.5 (0–4)) were tested with an extensive battery of neuropsychological tests near the time of MS diagnosis (average: 6 months). At the time of neuropsychological testing, 31 RRMS patients were still untreated, whereas 14 were treated with dymethilfumarate, 1 with fingolimod, 1 with natalizumab, 1 with interferon beta1-a, 1 with peg-interferon beta1-a, and 1 with azathioprine. Inclusion criteria for RRMS patients comprised diagnosis of RRMS [26], no relapse or steroid treatment in the 30 days before neuropsychological assessment, no concomitant neurological or other pathological health conditions, no substance abuse or other MS concomitant medication (as benzodiazepines or antidepressant drugs), and no visual impairment.

A group of 36 HCs, matched with RRMS patients for age, education, and gender, was recruited and tested with the same battery of neuropsychological tests used to assess RRMS patients. Inclusion criteria for HCs comprised no cognitive deficits measured with the Montreal Cognitive Assessment (MoCA) test [27], a test of global cognitive functioning; no neurologic, psychiatric, or other concomitant pathologies; normal or corrected to normal vision; no substance abuse or other prior or concomitant medications.

All participants were recruited at the MS Center of the Verona University Hospital (Verona, Italy). The study was approved by the local Ethics Committee, and written informed consent was collected from all participants. Demographic and clinical characteristics of RRMS and HCs are listed in Table 1.

### 2.2. Neuropsychological Assessment

RRMS patients and HCs were tested with an extensive battery of neuropsychological tests, which included the Brief Repeatable Battery (BRB) of neuropsychological tests [28]; the Stroop Test, ST [29]; the Phonological, Semantic, and Alternate Verbal Fluency test, (VF [30]); and the Modified Five Point Test (MFPT; [31]). The BRB is composed of tests of verbal learning and delayed memory recall (Selective Reminding Test, SRT); visuospatial learning and delayed memory recall (10/36 Spatial Recall Test, SPART); visual information processing speed and attention (Symbol Digit Modalities Test, SDMT); auditory information processing speed, attention, and calculation (Paced Auditory Serial Addition Task, PASAT); and semantic verbal fluency (Word List Generation, WLG). The ST is a test of attention and of automatic response inhibition; the VF test is a test of verbal fluency (phonemic, semantic, and alternate); the MFPT is a test of figurative fluency and use of strategies.

Depression, anxiety, and stress were evaluated with the 21-item Depression Anxiety Stress Scale (DASS-21; [32]) and subjective fatigue with the Fatigue Severity Scale (FSS; [33]). According to the most used method [6], RRMS patients were classified as “cognitive normal” (CN) if they scored below the cut-off (5° percentile; z-score = −1.65) on zero, one, or two neuropsychological tests administered; otherwise, if RRMS patients obtained a score below the cut-off on three or more neuropsychological tests, they were classified as having CI.

For each neuropsychological test and for each RRMS patient and HC, we calculated the Z-score index (for details see [34]), in which we did not use the normative data of the Italian validation of each test but, rather, the mean and standard deviation (SD) of scores of the RRMS patients and the HCs together. Considering the mean and SD of both groups together, in which MS patients and HCs compose the same population, allows to normalize the dependent variable (Z-score index) in a unique gaussian distribution with overlapped curves, mimicking a more real-life condition. Following this procedure, we calculated: (1) a global cognitive functioning index (Z-global) considering the average of the Z-scores of each neuropsychological test; and (2) three domain-specific Z-score indexes: memory (Z-MEM), attention/information processing speed (Z-ATT/IPS), and executive functions (Z-EF). For the detailed classification of each cognitive domain, see Table 2.

### 2.3. Statistical Analyses

ANOVA models with Tukey post-hoc analysis and chi-square test were applied to compare demographic, clinical, and Z-index scores among CI, CN, and HCs. Effects of EDSS, disease duration, emotional state (DASS-21), and fatigue (FSS) on the global cognitive functioning index (Z-score global index) and on the three cognitive domains (Z-MEM, Z-ATT/IPS, Z-EF) were controlled for RRMS patients’ by using a stepwise multiple regression analysis.

## 3. Results

Among the 50 RRMS patients tested, 14 were classified as having CI, and 36 as being CN. The majority (12/14: 86%) of the CI patients were impaired in the domains of ATT/IPS (64%) and EF (71%).

Group comparison results between CI (*n* = 14), CN (*n* = 36), and HCs (*n* = 36) showed no significant difference between the three groups in terms of age (*p* = 0.170), education (*p* = 0.229), and gender (*p* = 0.547).

**Figure 1:**
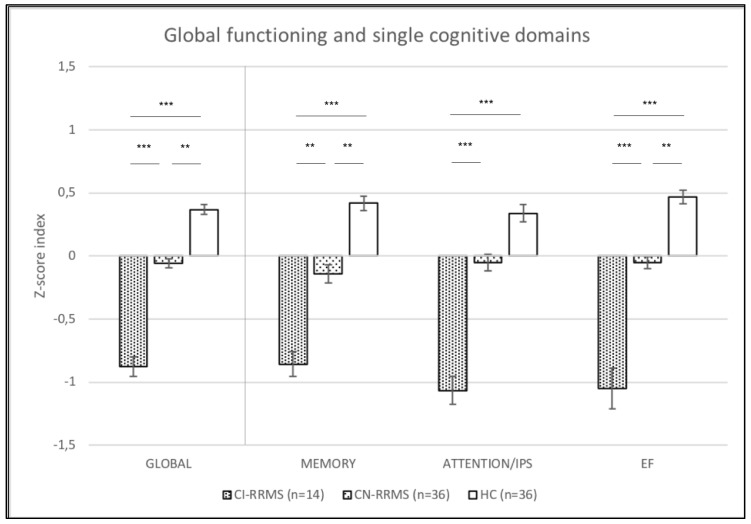
Functioning at a global level and on single cognitive domains of patients with cognitive impairment (CI), cognitive normal (CN) patients and healthy controls (HCs). ** = *p* < 0.01, *** = *p* < 0.001.

The Z-score global index was significantly different among the three groups (*p* < 0.001). Post-hoc comparisons showed a significant difference between CI and HCs (*p* < 0.001), between CI and CN (*p* < 0.001), and also between CN and HCs (*p* = 0.004) (Figure 1).

Significant difference was found among the three groups also for Z-MEM (*p* < 0.001), Z-ATT/IPS (*p* < 0.001), and Z-EFs (*p* < 0.001). Post-hoc analysis showed a significant difference between CI and HCs for Z- MEM (*p* < 0.001), Z-ATT/IPS (*p* < 0.001), and Z-EF (*p* < 0.001); between CI and CN for Z-MEM (*p* = 0.009), Z-ATT/IPS (*p* < 0.001), and Z-EF (*p* < 0.001); and between CN and HCs for Z-MEM (*p* = 0.005) and Z-EF (*p* = 0.006). No significant difference was found between CN and HCs for Z-ATT/IPS (*p* = 0.087), as shown in Figure 1.

Considering CI and CN patients together, the results of the multiple regression analysis (final model *R*^2^ = 0.254, *p* = 0.170) showed no significant effects of age (β = −0.255, *p* = 0.155), education (β = 0.196, *p* = 0.240), gender (β = 0.224, *p* = 0.185), disability (β = −0.125, *p* = 0.437), disease duration (β = 0.085, *p* = 0.591), emotional state (β = −0.197, *p* = 0.288), and fatigue (β = −0.145, *p* = 0.477) on the Z-global index. No significant effect of these variables was also found on Z-MEM (*R*^2^ = 0.289, *p* = 0.099), Z-ATT/IPS (*R*^2^ = 0.208, *p* = 0.311), and Z-EF (*R*^2^ = 0.252, *p* = 0.173).

Considering each single neuropsychological test, we found a significant difference among the three groups (CI, CN, and HCs) in all the neuropsychological tests (all *p* < 0.05), except for the WLG test (*p* = 0.180). Post-hoc analysis showed a significant difference between CI and HCs in all neuropsychological tests (all *p* < 0.05). Moreover, we found a significant difference between CI and CN for the SRT-CLTR (*p* = 0.013), SRT-D (*p* = 0.026), SDMT (*p* = 0.040), PASAT-3 (*p* = 0.001), PASAT-2 (*p* = 0.043), ST-Effect Interference Time (EIT) (*p* = 0.049), ST-Effect Interference Error (EIE) (*p* < 0.001), Phonemic Verbal Fluency (*p* = 0.023), Semantic Verbal Fluency (*p* = 0.042), MFPT-Unique Designs (UDs) (*p* = 0.001), and MFPT-Error Index (*p* = 0.020), as shown in Table 3. Finally, comparing CN and HCs, we found significant difference for the SRT-LTS (*p* = 0.012), SRT–CLTR (*p* = 0.016), SRT-D (*p* = 0.007), SDMT (*p* = 0.014), Phonemic Verbal Fluency (*p* = 0.034), MFPT-UDs (*p* = 0.003), and MFPT-Cumulative Strategies (CSs) (*p* = 0.019), as shown in Table 3.

## 4. Discussion

With the present study, we aimed at investigating the cognitive performance of a group of newly diagnosed RRMS patients as compared to a matched group of HCs by using a cognitive “functioning-based” approach instead of the classic “impairment-based” approach, in order to obtain a better real-life picture of RRMS patients’ effective cognitive functioning.

Considering a functioning-based approach (i.e., Z-score index), the results of the present study showed that newly diagnosed RRMS patients can differ significantly from a group of HCs both on a global level and with reference to the cognitive domains of attention/processing speed, memory, and executive functioning. However, the most interesting finding is related to the fact that this significant difference between RRMS patients and HCs persists even after isolating those patients classified as CN, considering the classic categorization criterion [6]. Specifically, the group of CN patients showed a significant decrease in cognitive performance as compared to HCs at the global level as well as in the domains of memory and executive functions. The grading scores assigned on the basis of this cognitive “functioning-based” approach, as opposed to the classic “impairment-based” approach, highlight that also newly diagnosed CN RRMS patients can show worse cognitive performance as compared to HCs since the early stages of the disease, independently of the effect of other clinical and demographical variables like age, education, physical disability, disease duration, fatigue, or emotional state. The classic cognitive “impairment-based” approach is undoubtedly affected by different cut-offs threshold and by the different number of neuropsychological tests used, which can render the diagnosis of CI uncertain. Given that cognitive decline can occur as a result of gradual progression related to neurodegeneration or of more transient changes related to inflammatory (i.e., relapses) disease activity, by using a functioning-based approach (i.e., Z-score index) we expected that also newly diagnosed RRMS patients would perform worse with respect to HCs. In fact, it has been found that brain alterations due to GM and WM lesions and inflammatory phenomena can be observed since the time of diagnosis and are related to differences in the inflammatory profile [35,36]. Considering previous studies that showed that early neurodegeneration phenomena affect mainly the frontal and the temporal lobes since the early stage of the disease [8], it is remarkable that a significant difference between newly diagnosed CN patients and HCs was found specifically in the domains of memory and executive functions, that are mainly related to the activity of frontal and temporal brain areas, respectively. We would like to strongly highlight the alterations in executive functioning, since this domain is often neglected and not included in the most used batteries of neuropsychological tests in MS (i.e., the BRB and the Brief International Cognitive Assessment for MS, BICAMS).

The Z-score index, in which cognitive performance is considered as a continuum, seemed to effectively reflect the accumulation of cognitive alterations even in those RRMS patients that would be classified as “cognitively normal”. As recently highlighted [37], if we accept that cognitive deficits in MS patients, or cognitive decline from baseline, reflect mainly cerebral dysfunctions related to MS disease, after excluding other confounding factors such as physical disability, fatigue, and emotional state, then cognitive functioning merits clinical attention as would any other indication of disease activity.

With this perspective, the classic impairment-based approach, usually limited by outdated and less representative normative data, can be overcome, optimizing the identification of slight alterations in cognitive performance already evident in newly diagnosed RRMS patients classified as being “cognitively normal” according to the traditional classification method. As underlined in previous studies, the early detection and monitoring of cognitive dysfunction may be crucial to identify MS patients with a probable worse prognosis and more severe disease progression [18,19], enabling early pharmacological and non-pharmacological interventions aimed at preventing further cognitive decline and disability in the long term [38]. According to this, a complete neuropsychological assessment in terms of level of performance, not just prone to classification criteria, seems of paramount importance not only in patients that show evident cognitive impairment [20], but also in apparently “cognitively normal” patients, as highlighted in the present study.

As recently underlined by Weber et al. [39], neuropsychological tests have shown a significant predictive value also regarding everyday-life activity and can be used in the clinical setting as one of several measures to help the clinician understand the impact of MS disease on the patients and their families. This view of considering patients’ “cognitive performance” instead of patients’ “cognitive impairment” might be an invaluable window on the real-life performance of MS patients since the time of diagnosis, given that early cognitive alterations can be considered as a signal of increased risk of disease progression [20].

We are aware that this study has some limitations. First, considering the variability of the MS population, further studies should include a larger number of both MS patients and matched HCs to substantiate the results of the present study. Second, the study is limited by the lack of a longitudinal neuropsychological assessment; this functioning-based approach should be tested more extensively with follow-up measures. Third, this study focused only on patients with RR course; future studies should extend this approach by investigating different MS populations. However, this is a proof-of-concept study, with which we aimed at highlighting the limitation of using the dichotomic approach derived from the classic neuropsychological assessment, frequently used for MS patients.

## 5. Conclusions

The results of the present study suggest that cognitive dysfunction in RRMS is a phenomenon that can be detected also in newly diagnosed patients. Extensive cognitive assessment since the early phase of the disease would be then of critical importance. This would support an accurate judgement of decline in cognitive functioning and would be clinically meaningful to determine a baseline cognitive profile to be monitored in the follow-up. We suggest approaching with extreme caution the traditional classification method of cognitive impairment: this classification criterion might fail in measuring the actual cognitive performance and should be interpreted with caution. In this regard, preferring an approach based on the evaluation of cognitive functioning as a continuous variable should be therefore recommended, also considering computerized devices [40,41].

## Figures and Tables

**Table 1:** Demographic and clinical characteristics of relapsing–remitting multiple sclerosis (RRMS) patients and healthy controls (HCs).

	CI (*n* = 14)	CN (*n* = 36)	HCs (*n* = 36)	*p*
Gender (M/F)	3/11	10/26	13/23	0.547
Age (years)	39.3 ± 14.0	37.8 ± 10.8	33.6 ± 10.4	0.170
Education (years)	13.8 ± 4.0	14.4 ± 2.0	15.1 ± 2.6	0.229
EDSS ^1^	2.0 (0–4)	1.0 (0–3)	/	/
Disease duration (years)	4.4 ± 8.2	3.1 ± 3.6	/	/
Time between diagnosis and neuropsychological assessment (months)	6 (±3)	6 (±2)	/	/

^1^ Means ± SDs were provided for continuous variables. Median (range) was provided for effects of disability (EDSS). EDSS = Expanded Disability Status Scale; CI = cognitive impairment; CN = cognitive normal.

**Table 2:** Neuropsychological tests considered for each Z-score domain index.

Z-MEM	Z-ATT/IPS	Z-EF
SRT-LTS	SDMT	ST (average EIT and EIE)
SRT-CLTR	PASAT-3	Phonemic VF
SRT-D	PASAT-2	Alternate VF
SPART-I		MFPT-UDs
SPART-D		MFPT-CSs

Z-MEM = Z-score–Memory; Z-ATT/IPS = Z-score–Attention/Information Processing Speed; Z-EF = Z-score–Executive Functions; SRT-LTS = Selective Reminding Test-Long-Term Storage; SRT-CLTR = Selective Reminding Test-Consistent Long-Term Retrieval; SRT-D = Selective Reminding Test-Delayed; SPART-I = Spatial Recall Test-Immediate; SPART-D = Spatial Recall Test-Delayed; SDMT = Symbol Digit Modalities Test; PASAT = Paced Auditory Serial Addition Test; ST-EIT = Stroop Test-Effect Interference Time; ST-EIE = Stroop Test-Effect Interference Error; VF = Verbal Fluency; MFPT-UDs = Modified Five Point Test-Unique Designs; MFPT-CSs = Modified Five Point Test-Cumulative Strategies.

**Table 3:** Neuropsychological performance of RRMS patients and HCs and results of the comparison between the Z-score indexes of each subtest. Means ± SDs are provided.

		CI (*n* = 14)		CN (*n* = 36)		HCs (*n* = 36)			
NP Battery/NP Test	Subtest	Raw Scores	Z-score Index	Raw Scores	Z-score Index	Raw Scores	Z-score Index	*p* CI vs. CN	*p* CN vs. HCs
BRB (Brief Repeatable Battery)	SRT-LTS	38.5 ± 15.7	−0.8 ± 1.2	47.3 ± 12.8	−0.2 ± 0.9	55.7 ± 10.2	0.5 ± 0.8	0.076	0.012 *
	SRT-CLTR	26.2 ± 13.1	−1.0 ± 0.8	39.9 ± 15.2	−0.1 ± 0.9	49.7 ± 14.3	0.5 ± 0.9	0.013 *	0.016 *
	SRT-D	6.8 ± 2.5	−0.9 ± 1	8.7 ± 2.5	−0.2 ± 1	10.3 ± 1.7	0.5 ± 0.7	0.026 *	0.007 *
	SPART	20.3 ± 4.4	−0.7 ± 1	22.7 ± 4.4	−0.1 ± 1	25 ± 4.1	0.4 ± 0.9	0.211	0.054
	SPART-D	6.9 ± 1.7	−0.6 ± 0.9	7.9 ± 2	−0.1 ± 1	8.8 ± 1.8	0.3 ± 0.9	0.246	0.145
	SDMT	45.1 ± 11.3	−0.8 ± 0.9	53.9 ± 9.7	−0.1 ± 0.8	61.7 ± 12.9	0.5 ± 1	0.04 *	0.014 *
	PASAT-3	31.2 ± 11.5	−1.1 ± 1	43.6 ± 10.6	0.009 ± 0.9	47.8 ± 9.6	0.4 ± 0.8	0.001*	0.198
	PASAT-2	26.1 ± 10.8	−0.9 ± 1.1	35.3 ± 9.9	0.009 ± 0.1	37.1 ± 8.7	0.2 ± 0.9	0.043 *	0.689
	WLG	23.8 ± 7	−0.5 ± 1.1	27.2 ± 6.6	0.06 ± 1	27.5 ± 6	0.1 ± 0.9	0.231	0.974
Stroop Test (ST)	ST-EIT	17.3 ± 7.8	−0.7 ± 1.4	13.4 ± 5.4	−0.04 ± 1	11.3 ± 3.5	0.3 ± 0.6	0.049 *	0.202
	ST-EIE	1.6 ± 2.3	−1.1 ± 2.1	0.2 ± 0.5	0.2 ± 0.4	0.1 ± 0.4	0.3 ± 0.3	0.000 *	0.882
Verbal Fluency Test (VF)	Phonemic	34.1 ± 11.9	−0.8 ± 0.9	44.7 ± 12.7	−0.01 ± 0.9	53.9 ± 10.6	0.7 ± 0.8	0.023 *	0.034 *
	Semantic	47.5 ± 11.5	−0.7 ± 1	56.2 ± 11.3	−0.005 ± 0.9	63.5 ± 9.1	0.6 ± 0.8	0.042 *	0.071
	Alternate	37.8 ± 12.3	−0.6 ± 1.1	43.2 ± 10.8	−0.09 ± 0.9	50.5 ± 9.9	0.5 ± 0.9	0.350	0.073
	Shifting Index	0.9 ± 0.3	0.06 ± 1.5	0.9 ± 0.1	−0.06 ± 0.9	0.9 ± 0.1	−0.02 ± 0.9	0.944	0.993
Modified Five Point Test (MFPT)	MFPT-UDs	23.2 ± 12.7	−1.1 ± 1.2	34.3 ± 7.7	−0.07 ± 0.7	41.9 ± 7.5	0.6 ± 0.7	0.001 *	0.003 *
	MFPT-CSs	8.7 ± 11.9	−0.7 ± 1	14.9 ± 10.6	−0.1 ± 0.9	22.8 ± 11.1	0.5 ± 0.9	0.193	0.019 *
	MFPT-Error Index	15.3 ± 16.8	−0.7 ± 1.7	6.7 ± 6.9	0.2 ± 0.7	7.1 ± 8.4	0.1 ± 0.8	0.02 *	0.985

* = significant result.
